# Fluid Behavior in Nanoporous Silica

**DOI:** 10.3389/fchem.2020.00734

**Published:** 2020-08-28

**Authors:** Salim Ok, Bohyun Hwang, Tingting Liu, Susan Welch, Julia M. Sheets, David R. Cole, Kao-Hsiang Liu, Chung-Yuan Mou

**Affiliations:** ^1^School of Earth Sciences, The Ohio State University, Columbus, OH, United States; ^2^Department of Chemistry, The Ohio State University, Columbus, OH, United States; ^3^Shull Wollan Center-A Joint Institute for Neutron Sciences, Oak Ridge National Laboratory, Oak Ridge, TN, United States; ^4^Department of Chemistry, National Taiwan University, Taipei, Taiwan

**Keywords:** low viscous fluids, confined state, relaxation, low-field NMR, subsurface

## Abstract

We investigate dynamics of water (H_2_O) and methanol (CH_3_OH and CH_3_OD) inside mesoporous silica materials with pore diameters of 4.0, 2.5, and 1.5 nm using low-field (LF) nuclear magnetic resonance (NMR) relaxometry. Experiments were conducted to test the effects of pore size, pore volume, type of fluid, fluid/solid ratio, and temperature on fluid dynamics. Longitudinal relaxation times (T_1_) and transverse relaxation times (T_2_) were obtained for the above systems. We observe an increasing deviation in confined fluid behavior compared to that of bulk fluid with decreasing fluid-to-solid ratio. Our results show that the surface area-to-volume ratio is a critical parameter compared to pore diameter in the relaxation dynamics of confined water. An increase in temperature for the range between 25 and 50°C studied did not influence T_2_ times of confined water significantly. However, when the temperature was increased, T_1_ times of water confined in both silica-2.5 nm and silica-1.5 nm increased, while those of water in silica-4.0 nm did not change. Reductions in both T_1_ and T_2_ values as a function of fluid-to-solid ratio were independent of confined fluid species studied here. The parameter T_1_/T_2_ indicates that H_2_O interacts more strongly with the pore walls of silica-4.0 nm than CH_3_OH and CH_3_OD.

## Introduction

There has been enormous interest in understanding the behavior of nanoconfined fluids due to its relevance in various areas such as biology and geochemistry (Vogel, 2010; Millischuk and Ladanyi, 2014). The behavior of molecules in confined geometries differs significantly from bulk behavior (Vogel, 2010; D'Agostino et al., 2012). This deviation arises from several factors including the relation between fluid and nanoporous matrix, and the effects of the size, shape, and geometry of the solid matrix on fluid behavior (Vogel, 2010; D'Agostino et al., 2012; Millischuk and Ladanyi, 2014). The issues on physical and chemical properties of confined fluids are heavily discussed (D'Agostino et al., 2012; Mallamace et al., 2014) along with the characterization of mesoporous solids with precise pore structure (Webber and Dore, 2004). Fundamental understanding of porous systems and their interaction with confined liquids is essential because mesoporous solids are used as model systems relevant to geological materials in the subsurface and have applications in separations, nanofluids, and catalysis (Millischuk and Ladanyi, 2014).

Mesoporous silica systems are a group of nanoporous materials with distinct cylindrical mesochannels, easily adjustable pore sizes, large surface areas, and even tunable particle sizes and shapes (Asefa and Tao, 2012). These mesoporous materials have generated interest because of their possible uses as supports for practical innovative materials (Al-Othman, 2012). Due to their large surface areas, these engineered proxies are ideal nanoporous systems for exploring confined fluid behavior at pore surfaces. For porous silica glasses, various degree of pore filling as compared to total pore volume of the nanoporous material with distilled water have been investigated with both relaxation and diffusion NMR approaches (Bhattachrja et al., 1989; D'Orazio et al., 1990a,b). Both longitudinal (T_1_) and transverse (T_2_) relaxation measurements showed a linear relationship with respect to fluid filling equivalent to monolayer coverage. This arises from the homogeneity of nanopores where the water molecules are evenly distributed. In the current study, we provide detailed characterization on the properties of mesoporous materials and discuss how the properties of these mesoporous materials influence the dynamics of confined fluids.

There are contradictory conclusions on the confined fluid behavior of nanoporous systems. For instance, D'Agostino et al. (2012) observed that diols, such as ethylene glycol and 1,2-propanediol, showed increased diffusivity within the pore space of titania (22 nm average pore size) and silica (13 nm average pore size) compared to alkanes including n-hexane and n-octane. T_1_ measurements also demonstrated that tumbling rate of polyols was not influenced by the porous medium while there was a significant drop of T_1_ for the alkanes. Among confined fluids, water has central significance for gaining insight into a wide range of systems including various geological and technological materials. In general, despite large number of literature work on confined water, the behaviors such as diffusion property of water in confined geometry is not fully understood (Ricci and Rovere, 2000; Swenson et al., 2001). The contradictory results are attributed to different factors such as competition between confinement and surface effects, dependence on temperature, and the characteristics of the surface interactions-hydrophilic vs. hydrophobic (Swenson et al., 2001). Experimental studies on water showed decreasing motion with increasing confinement for various surface substrates (Bellisent-Funel et al., 1995; Denisov and Halle, 1996; Zanotti et al., 1999). Contradictory results, such as an extensive hydrogen bonded network of water close to the surface (Steytler and Dore, 1985) vs. a reduction in number of hydrogen bonds per water molecule near cavity walls (Bruni et al., 1998) have been observed. Besides that, the influence of confinement is less pronounced in two-dimensional substrate type confinements than in three-dimensional confinements such as pores (Barut et al., 1998; Bergman and Swenson, 2000). Because of the controversial interpretations of the behaviors of confined fluids and water in particular, there is still a need to probe the molecular-level behavior of fluids as a function of a number of key parameters including, but not limited to, pore size, pore volume, fluid type, fluid-to-solid ratio, and temperature. In addition, conducting research on simple fluids such as water in confined state and developing models on confined behavior of low viscous fluids could be extended and utilized for better experimental design and understanding of complex fluids under confinement. For instance, mesoporous silica type materials are of special interest in crude oil industry, and these materials are ideal engineering proxies to investigate larger organic molecules such as decalin and tetradecane, and their mixtures to mimic a micro-environment resembling a petroleum aromatic fraction (Kapur et al., 2000) under confinement.

The novelty of the current work is studying dynamical behavior of fluids in confined states systematically by varying fluid volume, temperature, and pore diameter of the confining solid to better understand complex heterogeneous subsurface systems. These include fluids such as water and hydrocarbons in rock that have various wetting behaviors in the subsurface. The remarkable aspect of the mesoporous silica materials used in the present study is the utilization of them as catalyst materials in petroleum industry. Therefore, the projection of this work will be to extend the current efforts in terms of investigating more complex mixtures of fluids under confinement. In order to focus on that, first emphasis is showing distinguishability of confined vs. bulk-like fluids.

Our approach to distinguish the signals of fluids between confined and bulk-like states in different pore networks may have applications in rock core analysis using low-field NMR. To this end, in addition to using nanoporous silica powder, nanoporous silica rods (monolith samples) also were used, allowing for the study of confinement of water without excess water. Excess water has been observed in the case of mixtures of white powder nanoporous silica with water upon centrifugation, and it is bulk-like water. However, in the case of silica rods, there is no excess water outside the rod itself. First, the dynamical behaviors of bulk fluids in an NMR were determined by NMR relaxation measurements. Then the same NMR measurements were conducted on nanoporous silica powders having *confined* fluid (fluid in mesoporous silica and fluid interacting with the pore walls), as well as fluid in the interparticle regions of nanoporous silica powder and outside the pores, and *excess* fluid (fluid observed upon centrifugation of mixtures of nanoporous silica and fluids and showing bulk-like dynamical behavior). Hence, we aim to achieve the following goals: (i) to characterize the nanoporous silica systems in detail, (ii) to determine the degree of deviation of confined fluid behavior with respect to bulk fluid, (iii) to clarify the influence of pore parameters to the deviation of confined fluid behavior from bulk, and (iv) to show how to differentiate signals of confined fluid in nanopores from excess fluid.

## Materials and Methods

### Samples

Mesoporous silica with mean nanopore diameter and particle size of 4 and 200 nm, respectively, was purchased from Sigma-Aldrich. The silica porous monolith samples with nominal pore diameter of 5.0 nm, total pore volume of 0.7 cm^3^, specific density of 1.1 cm^3^, and BET surface area of 580 m^2^/g were purchased from Particle Solutions, LLC (Alachua, FL). Silica-1.5 nm was prepared by calcination of micellar template silica matrices made up of micrometer size grains. Pre-formed β-zeolite kernels (composed of tetraethylammonium hydroxide), NaOH, and fumed silica reacted with decylmethylammonium bromide solution in order to synthesize the silica matrix (Liu et al., 2013). The β-zeolite seeds were utilized to make the silica nanopore walls semi-crystalline and resilient to hydrolysis deterioration (Liu et al., 2000). The mixture was first relocated into an autoclave at 120°C for 2 days, then decreased to room temperature while adjusting the pH to 10. Upon sealing in an autoclave at 100°C for 2 days, the probe was accumulated by filtration in solid state, washed by water and ethanol, and dried at 60°C in air overnight. The ultimate mesoporous silica was obtained by calcination at 540°C for 8 h (Liu et al., 2013). Silica-2.5 nm was produced by an analogous procedure. The minor difference was the utilization of different carbon chain length surfactants or hydrothermal curing (Liu et al., 2013). Both silica-2.5 nm and silica-1.5 nm are cured with the second hydrothermal treatment; hence have even stronger structure and better hydrolysis resilience. Liu et al. (2006) provides further description on the synthesis of these two samples of silica-2.5 nm and silica-1.5 nm. It should be emphasized that the determined pore diameters of the samples in the present study are average values.

### Characterization

Pore size, pore volume, and surface area measurements were made with a Micromeritics ASAP 2020 gas sorption analyzer. The mesoporous silica samples were degassed at 423 K for 20 h under a vacuum pressure of 10 μm Hg to eliminate the impurities and gases within the pores. Nitrogen was the adsorbate used to acquire the adsorption and desorption isotherms at 77 K (see Table S1 and Figure S1 in Supporting Information). Transmission X-ray diffraction (TXRD) experiments were conducted with a PANalytical X'Pert Pro diffractometer. Transmission geometry permits accurate and precise measurement at the low 2θ angle range required for obtaining the long-range ordered pore structure. A thin layer of specimen was mounted between two films of Kapton foil to reduce beam absorption. TXRD measurements on nanoporous silica-4.0 nm and silica-1.5 nm were acquired using Cu Kα radiation and an X'Celerator detector. Data were acquired from 1 to 43° 2θ, with a step size of 0.02° 2θ and a speed of 20 s/step. Applied voltage and tube current for the measurements were 45 kV and 40 mA. TRXD measurement on silica-2.5 nm was acquired using a scintillation detector. Data were collected from 1 to 15° 25, with a step size of 0.02° 2. and a speed of 12 s/step. Applied voltage and tube current for the measurements were 45 kV and 40 mA (see Figures S2a–c) for TXRD scans of nanoporous silica samples).

Samples for thermogravimetric analysis (TGA) measurements were prepared by blending 150 mg of each silica sample with 1.0 ml distilled water in a 4 ml clear vial. The mixtures were kept at 20°C for a week for complete saturation of the pores with water. After centrifugation of the samples for 20 min at 5,000 rpm on an Eppendorf Centrifuge 5340 V 4.4, supernatant was taken. The samples were left again overnight after stirring with small glass rods. This procedure of centrifugation, removal of supernatant, and overnight keeping were repeated at least 10 days to make it sure that no excess water was left between the grains. Then water saturated porous silica samples were dried under continuous flow of neat air for durations ranging from 5 to 85 min. Successively, 10–20 mg samples were placed into a Pt crucible. Finally, the TGA measurements were run on a Perkin Elmer TGA7 Thermogravimetric Analyzer from 25 to 900°C under the flow of nitrogen gas with a flow rate of 15 ml/min and a heat rate of 20°C/min (see Figures S3a,b showing the TGA results). TG curves of silica samples prepared by different drying times exhibit weight loss behavior. The weight losses correspond to the elimination of water, and hence pore volume comparison of the mesoporous silica materials.

### Sample Preparation for Low-Field NMR Measurements

Hundred and fifty milligrams of each mesoporous silica sample were filled with three different amounts of distilled water 0.8, 0.6, and 0.4 ml. The samples were left overnight at least prior to conducting measurements for complete filling of the pores (see Figures S4a–e) in supporting information showing the photos of excess water after centrifugation and homogenization by sonication). For confining CH_3_OH and CH_3_OD, silica-4.0 nm was mixed with 0.9, 0.6, and 0.4 ml of each of these fluids. As with water, the samples were left overnight at minimum prior to conducting measurements to ensure complete filling of the pores. The samples were capped to prevent evaporation. Each sample was homogenized by sonication for 15 min immediately prior to the low-field NMR measurements. Controlled measurements were done to distinguish excess fluid signal from confined fluid signal (see Figures S4a–e). As seen in Figure S4, this was achieved by centrifugation of samples for 20 min at 5,000 rpm so that excess water migrated on top of the silica and water mixture (see Table 1 for the summary of the experimental conditions of the samples).

**Table 1:** Experimental conditions of the low-field NMR measurements.

**Measurements**	**Variable**	**Fluid type**	**Nanoporous matrix**	**Number of fluid/solid ratio**
T_1_	Pore diameter, temperature, fluid/solid ratio	H_2_O	Silica-4.0 nm; silica-2.5 nm; silica-1.5 nm	3
T_2_	Pore diameter, temperature, fluid/solid ratio	H_2_O	Silica-4.0 nm; silica-2.5 nm; silica-1.5 nm	3
T_1_	Pore diameter, temperature, fluid/solid ratio, fluid chemistry	CH_3_OH; H_2_O	Silica-4.0 nm	2
T_2_	Pore diameter, temperature, fluid/solid ratio, fluid chemistry	CH_3_OH; CH_3_OD	Silica-4.0 nm	3
T_1_, T_2_	Soaking time into water, temperature	H_2_O	Nanoporous silica rod-6.0 nm	2
**Measurements**	**Fluid/solid ratio**	**Temperature (K) (H**_**2**_**O)**	**Temperature (K) (CH**_**3**_**OH/CD**_**3**_**OD)**	**Figures**
T_1_	0.80 ml; 0.60 ml; 0.40 ml/0.150 g	298; 313; 323	-	2 (A–C)
T_2_	0.80 ml; 0.60 ml; 0.40 ml/0.150 g	298; 313; 323	-	3 (A–C)
T_1_, T_2_	0.60 ml; 0.40 ml/0.150 g	298; 313; 323	298; 313; 323	4 (A,B)
T_1_, T_2_	0.90 ml; 0.60 ml; 0.40 ml/0.150 g	-	298; 313; 323	5 (A,B)
T_1_, T_2_	10 and 90 min water soaking time	298; 313; 323	-	6 (A,B)

A second set of samples was prepared as follows: 218.0 mg silica-4.0 nm, 100.0 mg silica-2.5 nm, and 173.0 mg silica-1.5 nm were mixed with 0.4 ml deionized H_2_O. This adjustment was made to keep the pore volume of each nanoporous silica powder consistent around 120 cm^3^, for a given amount of surface area (see Table 2 for detailed values). The samples were left for soaking overnight at minimum prior to the measurements to ensure complete pore fillings. The samples were sonicated for 15 min to homogenize throughout the sample right before the measurements. The measurements were conducted at 313 K.

**Table 2:** T_1_ and T_2_ values of confined water obtained by keeping pore volumes approximately constant.

	**Silica-4.0 nm**	**Silica-2.5 nm**	**Silica-1.5 nm**
Amount (mg)	218.0	100.0	173.0
Total surface of given amount (m^2^)	130.1	116.7	143.9
Pore volume (cm^3^/g), single point at P/P_0_ = 0.99	1.04	0.97	1.20
Pore volume for given amount of surface area (cm^3^)	125.1	120.3	119.9
T_1_ (1) (ms)	500 ± 80	2,850 ± 60	2,670 ± 10
T_1_ (2) (ms)	270 ± 50	-	-
T_2_ (1) (ms)	19.6 ± 0.2	23.2 ± 0.4	18.5 ± 0.1
T_2_ (2) (ms)	-	70.0 ± 2.0	

*The measurements were conducted at 313 K*.

The third set of samples was prepared using porous silica monolith samples that were thermally treated overnight at 400°C to remove moisture and any organics left from the synthesis of the monolith samples. Upon cooling to 25°C, the samples were soaked in water for 10 min, measured and then soaked until 90 min. Visual inspection showed that the sample soaked for 10 min had non-transparent region in the middle of the sample, while such a region was not seen in the sample soaked for 90 min. This non-transparent region is attributed to volume not filled with water.

### ^1^H Low-Field NMR Relaxometry Measurements

Low-field NMR T_1_ and T_2_ relaxation measurements were performed on a Bruker Minispec mq20 NF Series instrument with a magnetic field strength of 0.47 T equivalent to a proton resonance frequency of 20 MHz (see Table 1 showing the details of experimental conditions), and the data were acquired utilizing Minispec software. The instrument contains a 10 mm temperature-variable probe. Temperature control is achieved using N_2_ flow and BVT temperature control unit. T_1_ measurements were completed using the inversion recovery pulse sequence found in the pulse sequence library of Bruker. T_2_ relaxation measurements were performed utilizing the standard Carr-Pucell-Meiboom-Gill (CPMG) pulse sequence, with τ of 1.0 ms between the 90 and 180° pulses. A continuous distribution of T_2_ exponential decays and T_1_ exponential growths correlated to confined and excess fluids were fitted for all T_2_ and T_1_ data using the CONTIN algorithm (Provencher, 1982). This analysis results in T_2_ and T_1_ distribution data. Bi-exponential decay and growth fittings of T_2_ and T1 curves, respectively, were completed using Origin 9.1 employing the following equations:

(1) $$\begin{array}{r} y=A_{21}e^{-x/T_{21}}+A_{22}e^{-x/T_{22}} \end{array}$$

(2) $$\begin{array}{r} y=A_{21}e^{x/T_{11}}+A_{22}e^{x/T_{12}} \end{array}$$

where T_21_, T_22_, T_11_, and T_12_ are the relaxation constituents, and A_21_ and A_22_ are the corresponding scales. The amplitudes A_21_ and A_22_ are directly proportional to the amount of fluid either in confined state or as excess. The fraction of T_21_ or T_11_ component is calculated as A_21_/(A_21_+A_22_) while portion of T_22_ or T_12_ component is determined as A_22_/(A_21_+A_22_).

## Results and Discussion

### Characterization of the Nanoporous Silica Materials

Figure S1 integrates the N_2_ adsorption and desorption isotherms for the three samples. Based on the shapes of the isotherms, the 2.5 and 4.0 nm samples can be classified as *Type IV* (Sing et al., 1985), which possesses mesoporous structure and poses the hysteresis loop caused by capillary condensation in mesopores (Thommes et al., 2015). The silica-2.5 nm material does not show strong hysteresis character because it is near the boundary of mesopore and micropore size ranges. The silica-1.5 nm isotherm is complicated by a *Type I* isotherm (micropore) below 0.8 P/P_0_, and a mesoporous hysteresis loop above 0.8 P/P_0_. The hysteresis is like the *H1* type (Tangestaninejad et al., 2009), which is composed of regular pores that have narrow pore distribution. The insets in Figure S1 show the pore size distributions for the three materials, where we can see that the silica-4.0 nm and silica-2.5 nm have uniform pore sizes, as determined by the Barrett-Joyner-Halenda (BJH) desorption and adsorption models, respectively. On the other hand, the silica-1.5 nm shows a bimodal pore distribution, as determined by density functional theory (DFT) model, where the dominant pore sizes are ~1.5 and ~2.5 nm (see Figure S1-inset).

Table S1 lists surface areas, volumes, and sizes of nanopores for the silica materials characterized for use in experiments. It needs to be mentioned that *meso*, which means “in between” in Greek, describes pore sizes ranging from 2.0 to 50.0 nm (Al-Othman, 2012; Thommes et al., 2015). In the present study, one of the silica samples, silica-1.5 nm, is out of the necessary range to be defined as *mesoporous*. This 1.5 nm material has higher surface area than the 4.0 nm material, contributed by the existence of micropores. In addition, the 2.5 nm material has relatively low pore volume, but the highest surface area measured, due to its nearly-microporous nature, and the highest surface-to-volume (S/V) ratio of the three porous silica systems. We will use the S/V ratio as a parameter for comparison in the discussion below.

Figure S2 shows TXRD scans for the three samples, with the contribution from kapton foil removed. The three peaks of silica-4.0 nm, shown in Figure S2a, can be indexed as (100), (110), and (200) reflections, respectively (Sarawade et al., 2013). A hexagonal mesostructure with an interplanar distance of 4.5 nm [d_(100)_] can be determined, and based on the hexagonal geometry, the sum of the pore wall and pore diameter is about 5.2 nm. This long range ordered structure is consistent with the single peak (~4.2 nm) in the pore size distribution plot (Figure S1) determined from gas sorption analysis. In addition, the TEM imaging of this nanoporous silica shows an ordered arrangement of about 4.0 nm pores in parallel (Ok et al., 2017).

Figure S2b shows the five diffraction maxima observed in the TXRD scan of silica-2.5 nm, with *d*-spacings 3.1, 2.1, 1.9, 1.5, and 1.3 nm, respectively. According to their ratios, they can be indexed as (110), (200), (211), (310), and (222) reflections, and this ordered structure belongs to the cubic *Im3m* space group. Therefore, the sum of the pore wall and the pore diameter is ~4.2 nm, indicating that the pore wall is relatively thick, considering the pore diameter of 2.8 nm as determined by the BJH adsorption model. Figure S2c shows the silica-1.5 nm TXRD scan with diffraction maxima corresponding to *d*-spacings 2.2 and 1.5 nm. Although the specific pore structure arrangement is not identified in this case, these measurements, along with the pore distribution plot for silica-1.5 nm (Figure S1), suggest that it may have two dominant pore dimensions.

Pore volume is directly related to the amount of confined fluid. As demonstrated in Figure S3a, all of the porous silica samples where water was confined showed one-step mass loss. However, the samples with shorter drying times had fractions of water that persist till higher temperatures such as 200°C, as shown in Figure S3a, demonstrating representative weight loss in the case of silica-4.0 nm. As the sample-drying time was increased, complete weight-loss temperature shifted toward 95°C. In other words, all the water weight loss only required heating 95°C when the sample was dried for longer period of time. Similar results were observed when water was confined into silica samples with 2.5 and 1.5 nm pore diameters. At temperatures higher than 200°C no weight-loss occurred, and this shows that mesopores may intensely restrict water molecules and offer media for both thermodynamic and kinetic barrier to the elimination of water molecules (Wu and Navrotsky, 2013). These effects may resemble the consequences of confinement in geological environments with widely-ranging pore sizes. As shown in Figure S3b, the weight loss of water was less in silica-2.5 nm than for both silica-4.0 nm and silica-1.5 nm at the end of 12 min of drying time. This clearly showed that silica-2.5 nm has lower pore volume than the other two-engineered proxies of interest. Comparing the mass loss at the end of 20 min drying, we suggest that silica-1.5 nm has higher volume than that of silica-4.0 nm.

### Excess Fluid vs. Confined Fluid

**Figure 1:**
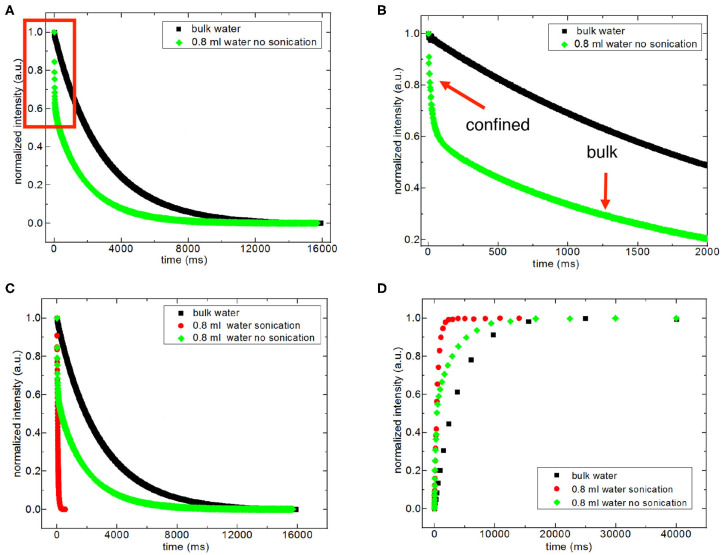
Representative comparison of T_2_ data of 0.8 ml water in the mixture with 150 mg silica-4.0 nm (S/V = 574) with and without sonication acquired at 40°C **(A–C)**. **(B)** is the zoom in for the red box in **(A)**. Representative comparison of T_1_ data of 0.8 ml water in 150 mg silica-4.0 nm with and without sonication acquired at 40°C **(D)**.

For discriminating excess water signal from signal of water confined into nanoporous silica samples, first T_1_ (inversion recovery) and T_2_ (CPMG) measurements at 40°C on the low-field NMR instrument were conducted. Figure S4a shows excess water showing bulk-like behavior on top of the mixture of silica-4.0 nm and confined water. As seen in Figure 1 and Table 3A, it is possible to differentiate excess water signal from that of confined water. T_1_ time values of bulk like water on top of the mixture without sonication are closer to that of water in bulk, while T_1_ values of confined water signal is lower than that of T_1_ values of confined water in the case of homogenized sample by sonication. This also exhibits that T_1_ values measured by homogenizing (sonication) are average values of water with two different environments: in between the grains and in the mesopores. Buntkowsky et al. (2007) mentioned that the sticking together is mainly the consequence of H_2_O molecules high polarity and their capability to establish hydrogen bond networks among the water molecules. However, dynamics of water become more complicated in confined geometries due to opposition between the surface-liquid and liquid-liquid relations. This opposition forms new structures of water as in the case of partial ordering water molecules in the neighborhood of the restraining surface.

**Table 3A:** T_1_ and T_2_ values of confined water measured on the 20 MHz instrument at 40°C with either excess water on top or after homogenization with sonication.

**Sample**	**T_**2**_ (ms)**	**T_**1**_ (ms)**	**Experimental treatment**
Bulk water	2,883.3 ± 0.5	3,960 ± 20	-
150 mg silica-4.0 nm + 0.8 ml H_2_O	(1): 50.48 ± 0.08	(1): 478 ± 6	After sonication
150 mg silica-4.0 nm + 0.8 ml H_2_O	(1): 34.5 ± 0.2	***(1): 200 ± 8***	Without sonication/with excess fluid
	(2): 1,956 ± 1	***(2): 3,300 ± 100***	
150 mg silica-4.0 nm + 0.6 ml H_2_O	(1): 36.48 ± 0.06	(1): 350 ± 10	After sonication
		(2): 470 ± 20	
150 mg silica-4.0 nm + 0.6 ml H_2_O	(1): 29.4 ± 0.1	***(1): 201 ± 87***	Without sonication/with excess fluid
	(2): 2,315 ± 1	***(2): 3,500 ± 100***	
150 mg silica-2.5 nm + 0.8 ml H_2_O	(1): 24.2 ± 0.3	(1): 2,830 ± 20	After sonication
	(2): 91.0 ± 3.0		
150 mg silica-2.5 nm + 0.8 ml H_2_O	(1): 15 ± 2.0	(1): 2,760 ± 20	Without sonication/with excess fluid
	(2): 38.6 ± 0.7		
150 mg silica-2.5 nm + 0.6 ml H_2_O	(1): 17.0 ± 5.0	(1): 2,660 ± 20	After sonication
150 mg silica-2.5 nm + 0.6 ml H_2_O	(1): 17.57 ± 0.10	(1): 70 ± 20	Without sonication/with excess fluid
		(2): 2,600 ± 10	
150 mg silica-1.5 nm + 0.8 ml H_2_O	(1): 9.6 ± 0.2	(1): 2,200 ± 200	After sonication
	(2): 55.1 ± 0.6	(2): 1,100 ± 200	
150 mg silica-1.5 nm + 0.8 ml H_2_O	(1): 5.74 ± 0.03	**(1): 110 ± 2**	Without sonication/with excess fluid
	(2): 1,406.9 ± 0.4	**(2): 2,800 ± 100**	
150 mg silica-1.5 nm + 0.6 ml H_2_O	(1): 5.1 ± 0.5	(1): 1,000 ± 50	After sonication
	(2): 41.7 ± 0.1	(2): 2,100 ± 100	
150 mg silica-1.5 nm + 0.6 ml H_2_O	(1): 16.7 ± 0.10	**(1): 680 ± 20**	Without sonication/with excess fluid
	(2): 927.1 ± 0.6	**(2): 3,500 ± 10**	

*Bold and italic values are with excess fluid on top. In particular, T_1_(2) are the values closer to that of bulk fluid*.

The other approach of the T_2_ data analysis belonging to the samples without sonication and with excess water on top (see Figures S4a,d) is determination of the percentages of bulk like water and confined water based on the Equation (1) as suggested in the literature (Aursand et al., 2008). This is a simple and robust technique to evaluate the T_2_ relaxation data. At first we compare the results of bi-exponential fitting of the data belonging to water mixtures with either silica-4.0 nm or silica-1.5 nm having bulk-like water on top as shown in Figures S4a,d. As seen in Table 3A, longer T_2_ values are assigned to bulk-like water. The percentage of bulk-like water on top of the mixture is not ~ <60% (Table 3B). Then in this case, nearly 40% of H_2_O is in between grains and confined establishing a dynamic system. Referring to Table S1, it is possible to calculate the pore volume given that the mass of the nanoporous silica is known. Thus, we can differentiate the volume of confined water from the volume of water in between the grains.

**Table 3B:** Quantification of confined vs. excess water.

**Sample**	**Treatment**	**A_**21**_ (longer T_**2**_)**	**A_**22**_ (shorter T_**2**_)**	**Amount of H_**2**_O on top (ml)**	**The rest of H_**2**_O (ml)^a^**	**Amount of H_**2**_O between grains (ml)^b^**	**Confined H_**2**_O (ml)^c^**	**Pore volume (ml)^d^**
150 mg silica-4.0 nm + 0.8 ml H_2_O	Centrifuge/with excess fluid	0.61	0.39	0.49	0.31	0.16	0.16	0.16
150 mg silica-4.0 nm + 0.6 ml H_2_O	Centrifuge/with excess fluid	0.63	0.38	0.38	0.23	0.07	0.16	0.16
150 mg silica-1.5 nm + 0.8 ml H_2_O	Centrifuge/with excess fluid	0.53	0.47	0.42	0.38	0.20	0.18	0.18
150 mg silica-1.5 nm + 0.6 ml H_2_O	Centrifuge/with excess fluid	0.60	0.40	0.36	0.24	0.06	0.18	0.18

a *Volume of water confined and between grains*.

b *Difference between 1 and 3*.

c *Volume of confined water which is equal to pore volume*.

d *Pore volume determined by using density data in Table S1*.

In the case of homogenized samples by sonication, because the nanoporous silica materials of interest in the current study have the same structure of MCM-41, we refer to the proposed filling mechanism of MCM-41 having 4.6 nm of pore-to-pore distance by water (Grünberg et al., 2004). Water was studied as a guest molecule in mesoporous silica, MCM-41 and SBA-15, with two-dimensional hexagonally arranged of cylindrical pores in identical size ranging from 2 to 10 nm. Due to the high density of pores and relatively small pore diameters, these silica materials have bigger inner surfaces with respect to the volume of the single particle. There is a favored axis present in the direction of the pores cylinder axis arising from highly anisotropic geometry of the pores. In MCM-41, following the first wetting of the pore surfaces, a co-presence of filled pores or partially filled pores occurs. Additional filling of the pores happens as an enlargement of the filled pores till whole filling is obtained once more. Therefore, for MCM-41 the water layer grows axially in the direction of the pore axis. In the case of 0.4 ml H_2_O wetted silica-4.0 nm or silica-1.5 nm there were initially wetted pore surfaces, filled pores and not wetted pore (dry) segments coexisted. In mixtures of nanoporous silica samples mixed with either 0.6 or 0.8 ml H_2_O, after homogenization by 15 min sonication, the confined water molecules were in rapid “conversation” with the excess water molecules occupying the space in between the grains. The water molecules inside the nanoporous silica did the fast exchange with the excess water molecules by moving axially in the direction of the pores. On the NMR time scale, average T_1_ or T_2_ values were obtained. These average relaxation times are shorter than not only these of bulk water but also bulk-like water on top of the silica and water mixtures. There was no apparent bulk-like water in the case of mixtures with 0.4 ml water. For this reason, we did not apply bi-exponential fitting analysis for these samples having the lowest fluid-to-solid ratio.

### Dynamics of Confined Fluids

**Figure 2:**
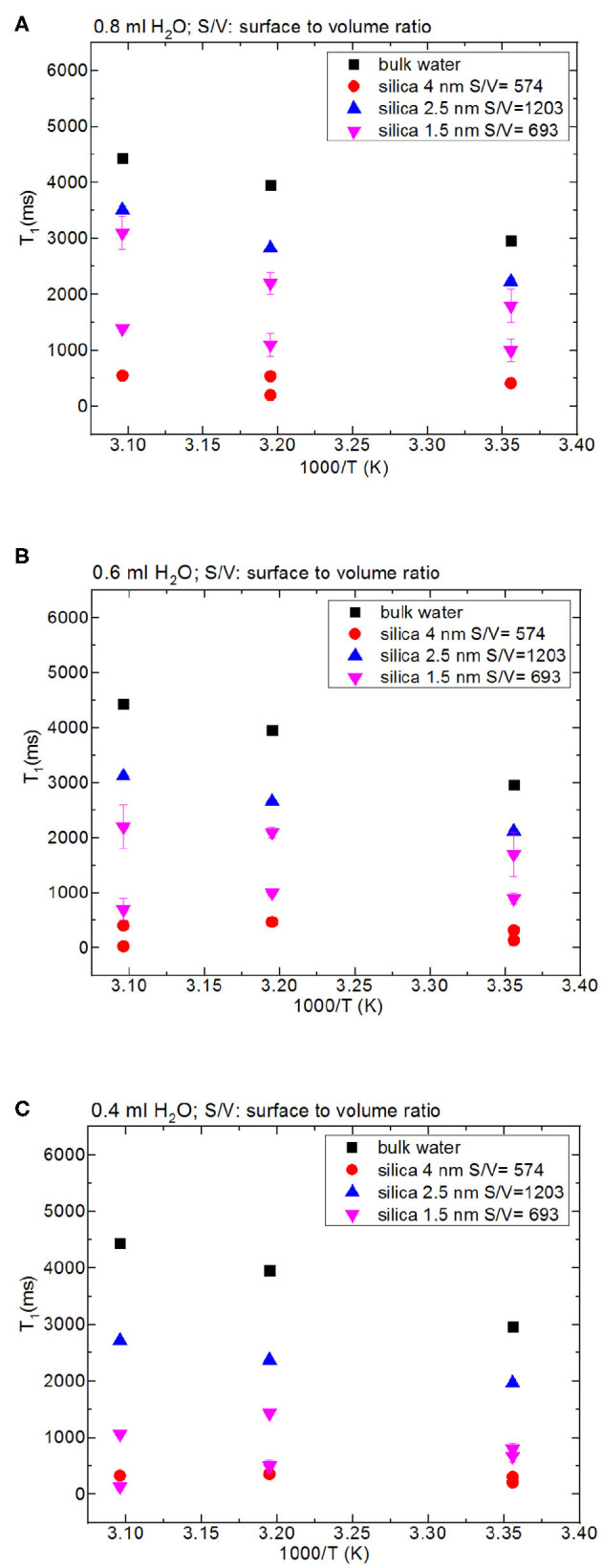
Comparison of T_1_ values of bulk water and water mixed with nanoporous silica where the variables are pore diameter of nanoporous silica with the amount of 150 mg (silica-4.0 nm, silica-2.5 nm, silica-1.5 nm), temperature (298, 313, 323 K) and volume of water [0.8 ml water **(A)**, 0.6 ml water **(B)**, 0.4 ml water **(C)**].

Then we focus on dynamical behaviors of confined fluids by varying temperature, fluid-to-solid ratio, and pore diameter. Table 4A lists T_1_ values of bulk and confined water. As seen in Figures 2A–C, deviation and change of confined fluid behavior from that of bulk fluid is independent of pore size of the nanoporous matrix systems. At the fluid-to-solid ratios of 0.8, 0.6, and 0.4 ml to 150 mg silica-2.5 nm, T_1_ times increased as the temperature was increased. However, in the case of fluid-to-solid ratios of 0.8, 0.6, and 0.4 ml to 150 mg silica-4.0 nm, T_1_ times did not show significant change when temperature was varied. When 0.8 ml water was confined to 150 mg silica-1.5 nm, as the temperature was increased, T_1_ times also increased. However, with the fluid-to-solid ratios of 0.6 and 0.4 ml water to 150 mg silica-1.5 nm, first there was an increase in T_1_ time as the temperature was increased, but when the temperature was increased further, T_1_ time decreased. Sattig et al. (2014) studied temperature-dependent rotational motion of super-cooled H_2_O in MCM-41 type silica pores of diameters 2.93, 2.76, and 2.14 nm using ^2^H NMR. There was a first sharp twist observed in the temperature reliance escorted by a solidification of a portion of the confined H_2_O. This implied an alteration from bulk-like to interface-dominated water dynamics instead of a liquid-liquid phase changeover. In the temperature range above 225 K, there was the confinement effect observed. Above 225 K, the temperature reliance of H_2_O re-location was weaker in the smaller pores, and in the bigger pores bulk-like water behavior was seen. Near 225 K, longitudinal magnetization relaxation (T_1_) times for the ice in silica-2.1 nm confinement became very long for a dependable determination within a sensible duration, while T_1_ times for confined water in liquid state passes a minimum, showing that confined H_2_O has correlation times τ ≈ 1/ω_0_ ≈ 1 ns. It was suggested that ^2^H NMR line-shape analysis evidenced pronounced dynamical heterogeneities for confined H_2_O. However, in our study we did not conduct line-shape analysis; rather focus on T_1_ measurements at high temperatures. Temperature range of the study was out of interest of the current contribution. However, Sattig et al. (2014) mentioned that the temperature reliance of water re-location is largely independent of the confinement dimensions. We observe a similar result that deviation of confined water behavior from that of bulk water is independent of pore diameter, while degree of confinement effect on dynamics of confined water is more pronounced in the case of silica-4.0 nm. This is reflected in the T_1_ values as follows: the longest T_1_ values were observed when water was confined to silica-2.5 nm, while the shortest T_1_ values were seen when water was confined to silica-4.0 nm. We explain this situation with surface-to-volume (S/V) ratios of the nanoporous silica materials rather than pore diameter. Timur (1969) claimed that in a three-component NMR model, the pore volumes of a porous medium were classified as three sub-groups, based on their S/V ratio distribution. He explained that the longer T_1_ times would correspond to the smaller S/V ratios, and the larger pores. In our case, the T_1_ times do not show systematic change as a function of pore diameter. Rather, T_1_ times become longer when water molecules are confined into silica-2.5 nm with the highest S/V ratio, while the shortest T_1_ times are observed upon confining H_2_O molecules into silica-4.0 nm with the lowest S/V ratio. For this reason, the trend in T_1_ times of water confined into silica materials is attributed to the S/V ratios rather than pore diameter.

**Table 4A:** Longitudinal magnetization relaxation times (T_1_) of confined water obtained after sonication of the mixtures for 15 min.

	**Temperature (°C)**		
**Samples**	**25°C**	**40°C**	**50°C**
Bulk H_2_O	2,960 ± 20 ms	3,960 ± 20 ms	4,440 ± 50 ms
150 mg silica-4.0 nm + 0.8 ml H_2_O	(1): 413 ± 30 ms	(1): 478 ± 6 ms	(1): 550 ± 10 ms
150 mg silica-4.0 nm + 0.6 ml H_2_O	(1): 270 ± 8 ms	(1): 350 ± 10 ms	(1): 405 ± 3 ms
	(2): 360 ± 10 ms	(2): 470 ± 20 ms	
150 mg silica-4.0 nm + 0.4 ml H_2_O	(1): 210 ± 20 ms	(1): 254 ± 9 ms	(1): 30 ± 6 ms
	(2): 310 ± 30 ms	(2): 400 ± 10 ms	(2): 329 ± 5 ms
150 mg silica-2.5 nm + 0.8 ml H_2_O	(1): 2,230 ± 20 ms	(1): 2,830 ± 20 ms	(1): 3,510 ± 80 ms
150 mg silica-2.5 nm + 0.6 ml H_2_O	(1): 2,120 ± 20 ms	(1): 2,660 ± 20 ms	(1): 3,120 ± 20 ms
150 mg silica-2.5 nm + 0.4 ml H_2_O	(1): 1,970 ± 60 ms	(1): 2,370 ± 10 ms	(1): 2,720 ± 30 ms
150 mg silica-1.5 nm + 0.8 ml H_2_O	(1): 1,000 ± 200 ms	(1): 1,100 ± 200 ms	(1): 1,390 ± 70 ms
	(2): 1,800 ± 300 ms	(2): 2,200 ± 200 ms	(2): 3,100 ± 300 ms
150 mg silica-1.5 nm + 0.6 ml H_2_O	(1): 900 ± 100 ms	(1): 1,000 ± 50 ms	(1): 700 ± 200 ms
	(2): 1,700 ± 400 ms	(2): 2,100 ± 100 ms	(2): 2,200 ± 400 ms
150 mg silica-1.5 nm + 0.4 ml H_2_O	(1): 670 ± 100 ms	(1): 500 ± 100 ms	(1): 130 ± 40 ms
	(2): 800 ± 100 ms	(2): 1,440 ± 40 ms	(2): 1,060 ± 10 ms

**Figure 3:**
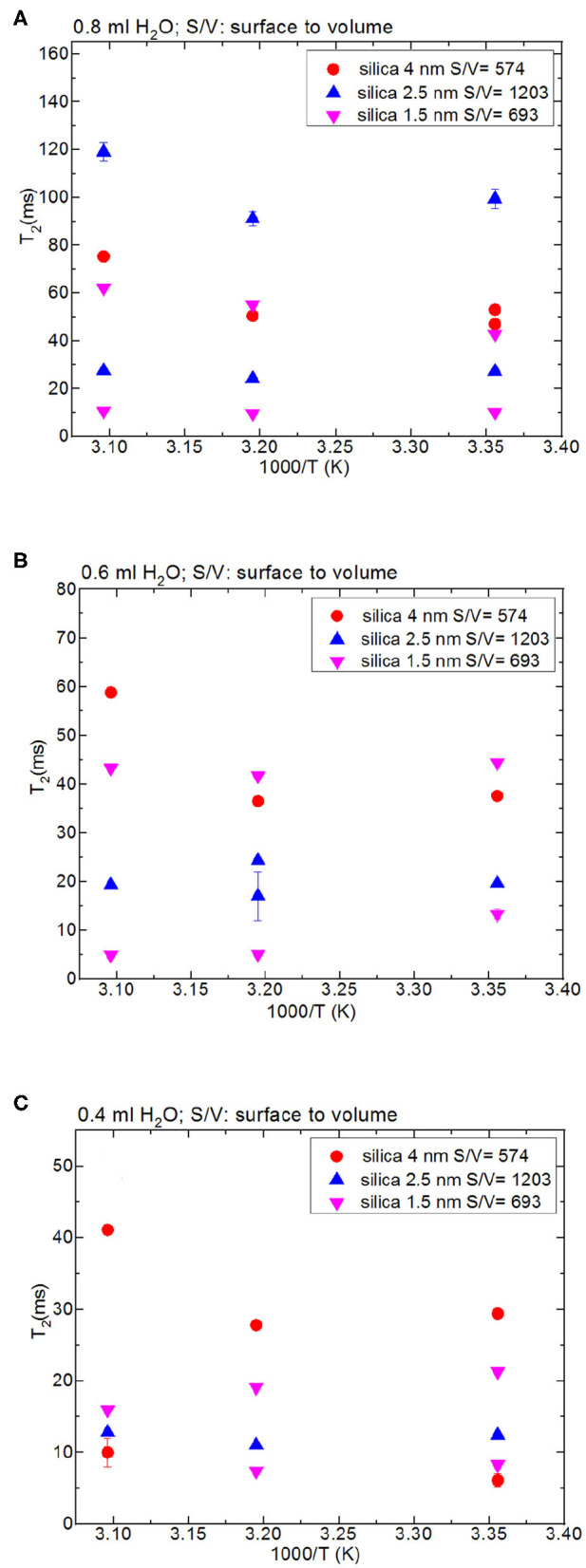
Comparison of T_2_ values of bulk water and water mixed with nanoporous silica where the variables are pore diameter nanoporous silica materials with the amount 150 mg (silica-4.0 nm, silica-2.5 nm, silica-1.5 nm) and temperature (298, 313, and 323 K); 0.8 ml water **(A)**, 0.6 ml water **(B)**, 0.4 ml water **(C)**.

Besides analyzing T_1_ times, T_2_ times of the confined fluids were also analyzed. Table 4B lists the T_2_ results. As seen in Figures 3A–C, when the fluid-to-solid ratio is decreased, T_2_ values also decrease. This is independent of the pore diameter of the nanoporous silica materials. T_2_ values in the case of the fluid-to-solid ratio of 0.4 ml to 150 mg silica (partial filling of the pores) are longer for silica-4.0 nm than silica-1.5 nm and silica-2.5 nm. In the case of 0.6 ml fluid to 150 mg silica ratio, T_2_ values of water confined into silica-4.0 nm and silica-1.5 nm are closer to each other, and longer than confined into silica-2.5 nm. With the highest fluid-to-solid ratio, the longest T_2_ values are observed when water is confined into silica-2.5 nm. Having smaller fluid-to-solid ratio means filling the pores with lower volume easily. Because silica-2.5 nm has lower pore volume as shown by TGA measurements (see Figures S3a,b), in the case of the lowest fluid-to-solid ratio “majority” of the fluid molecules completely fill the pores. This in turn is indicated with shorter T_2_ values. There is an important trend in T_2_ values. As the fluid-to-solid ratio is decreased, T_2_ values also decrease. However, the decrease is more effective in the case of silica-2.5 nm, which has the lowest pore volume.

**Table 4B:** Transverse magnetization relaxation times (T_2_) of confined water obtained after sonication of the mixtures for 15 min.

	**Temperature (°C)**		
**Samples**	**25°C**	**40°C**	**50°C**
Bulk H_2_O	2,440 ± 0.2	2,883.3 ± 0.5	3,122.2 ± 0.2
150 mg silica-4.0 nm + 0.8 ml H_2_O	(1): 47.0 ± 2.0 ms	(1): 50.5 ± 0.1 ms	(1): 75.3 ± 0.1 ms
	(2): 53.0 ± 2.0 ms		
150 mg silica-4.0 nm + 0.6 ml H_2_O	(1): 37.6 ± 0.1 ms	(1): 36.5 ± 0.1 ms	(1): 58.8 ± 0.1 ms
150 mg silica-4.0 nm + 0.4 ml H_2_O	(1): 29.4 ± 0.2 ms	(1): 27.8 ± 0.1 ms	(1): 44.3 ± 0.6 ms
	(2): 6.1 ± 0.9 ms		(2): 14.0 ± 2.0 ms
150 mg silica-2.5 nm + 0.8 ml H_2_O	(1): 27.2 ± 0.4 ms	(1): 24.2 ± 0.3 ms	(1): 27.5 ± 0.2 ms
	(2): 99.0 ± 4.0 ms	(2): 91.0 ± 3.0 ms	(2): 119 ± 4.0 ms
150 mg silica-2.5 nm + 0.6 ml H_2_O	(1): 19.7 ± 0.1 ms	(1): 17.0 ± 5.0 ms	(1): 19.3 ± 0.1 ms
150 mg silica-2.5 nm + 0.4 ml H_2_O	(1): 12.5 ± 0.1 ms	(1): 11.0 ± 0.1 ms	(1): 12.8 ± 0.1 ms
		(2): 24.3 ± 0.4 ms	
150 mg silica-1.5 nm + 0.8 ml H_2_O	(1): 10.1 ± 0.5 ms	(1): 9.6 ± 0.2 ms	(1): 10.7 ± 0.2 ms
	(2): 42.8 ± 0.3 ms	(2): 55.1 ± 0.6 ms	(2): 62.2 ± 0.3 ms
150 mg silica-1.5 nm + 0.6 ml H_2_O	(1): 13.3 ± 1.0 ms	(1): 5.1 ± 0.5 ms	(1): 4.9 ± 0.5 ms
	(2): 44.4 ± 0.5 ms	(2): 41.7 ± 0.1 ms	(2): 43.3 ± 0.1 ms
150 mg silica-1.5 nm + 0.4 ml H_2_O	(1): 8.3 ± 0.5 ms	(1): 7.4 ± 0.5 ms	(1): 15.9 ± 0.1 ms
	(2): 21.3 ± 0.3 ms	(2): 19.1 ± 0.3 ms	

The other set of measurements was conducted by keeping the pore volume of each nanoporous silica constant around 120 cm^3^ (see Table 2). Among the three silica samples, silica-2.5 nm has the highest S/V ratio. When the pore volume is kept constant at ~120 cm^3^, water has the longest T_1_ value in the mixture with silica-2.5 nm. Similarly, the highest T_2_ was obtained for the same water and silica-2.5 nm mixture. The T_1_ value of water in the mixture with silica-1.5 nm is slightly lower than that of water in the mixture with silica-2.5 nm. The results on relaxation of water obtained by keeping pore volumes of porous matrixes constant for the given amounts of the porous materials indicate clearly that pore diameter does not affect the dynamics of confined water significantly. Rather, S/V ratio along with pore diameter reflects information on filling mechanism of nanoporous silica materials with water. In particular, when the pore diameter is big and S/V ratio is small, at first water molecules wet the surface of the pore walls. Additional filling happens easily from the pore wall to the midpoint of the pore. Then complete filling of the pores is achieved (Grünberg et al., 2004). In the case of nanoporous silica with small pore diameter and large S/V ratio, the filling mechanism occurs in a different way: first the center is filled; hence there is a co-presence of filled pore fragments with wetted pores. Further filling of the pores develops axially in the direction of the pore axis. The pore volume is also related to pore length when we assume cyclindrical pores. The nanoporous silica with small pore diameter and long pore axis leads to larger pore volumes. The long pore length (large pore volume) gives enough degree of freedom for confined water molecules. Relatively free confined water molecules prefer motion in axial direction, and this results in weaker interactions with pore walls. This is a possible explanation of larger T_1_ values for pores with smaller pore diameter and larger pore volume.

**Figure 4:**
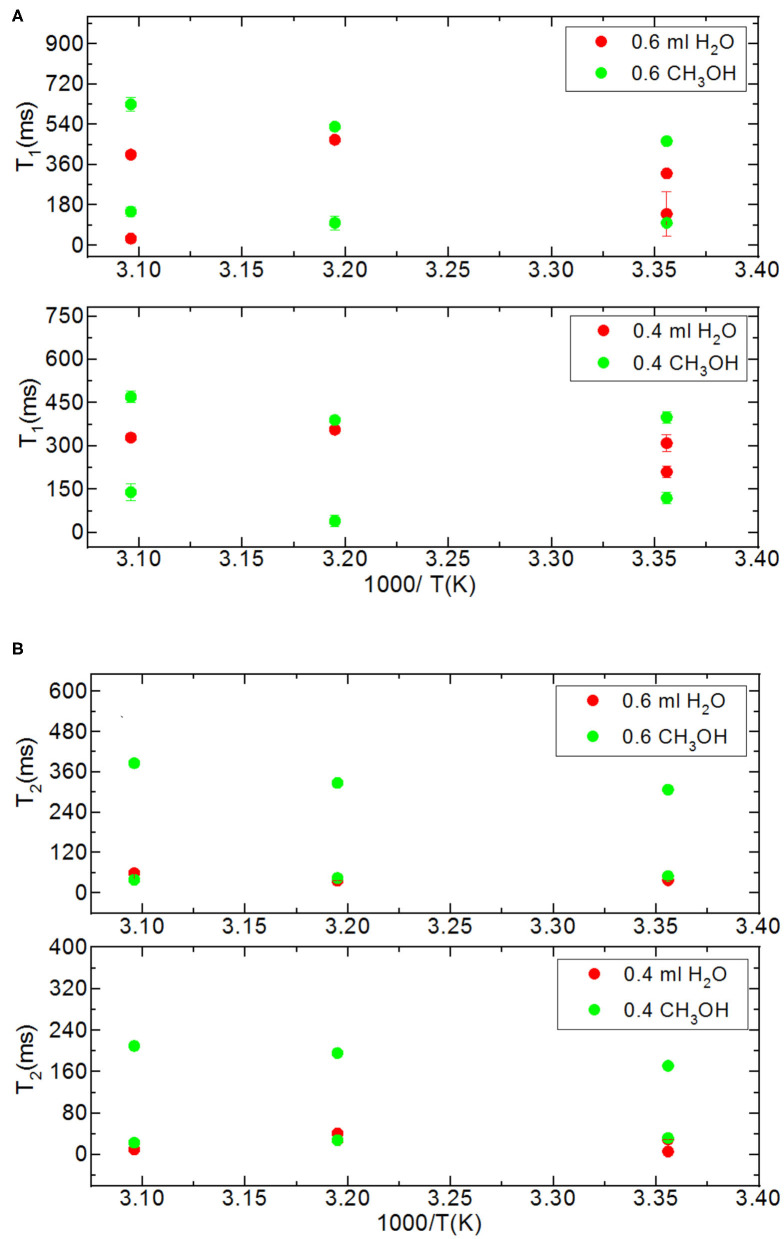
Comparison of relaxation values of H_2_O and CH_3_OH confined into silica-4.0 nm with the amount of 150 mg and S/V = 574 where the variables are temperature values (298, 313, and 323 K), and fluid-to-solid ratios (0.60 ml/150 mg, 0.40 ml/150 mg); T_1_ **(A)** and T_2_ **(B)**.

In addition to the effect of pore volume and S/V ratios of the porous systems on dynamical behaviors of confined H_2_O, fluid chemistry is also studied by confining CH_3_OH and CH_3_OD into nanoporous silica-4.0 nm. Tables 5A,B list T_1_ and T_2_ values of bulk and confined CH_3_OH and CH_3_OD. First, T_1_ and T_2_ relaxation times of confined methanol deviated from those of bulk methanol. The second remarkable point is the higher T_1_ and T_2_ values of CH_3_OD with respect of those of CH_3_OH in confined state. Internal rotation of the –CH_3_ group is faster than the –OD group rotation in turn leading to longer T_1_ times of CH_3_OD compared to that of CH_3_OH. As seen in Figures 4A,B, confined methanol (CH_3_OH) has slightly higher T_1_ relaxation values than these of confined H_2_O. This result is independent of fluid-to-solid ratio. This is also related to the faster internal rotation of –CH_3_ group. As expected, when the fluid-to-solid ratio is decreased, T_1_ values of confined methanol also decreased. Methanol has two T_1_ values in confined state. In the case of higher fluid-to-solid ratio, longer T_1_ increases as temperature increases, while shorter T_1_ does not show significant change upon elevating temperature. In the case of lower fluid-to-solid ratio for both longer and shorter T_1_ values, T_1_ minimum is observed as a function of temperature.

**Table 5A:** Longitudinal magnetization relaxation times (T_1_) of confined CH_3_OH and CH_3_OD obtained after sonication of the mixtures for 15 min.

	**Temperature (°C)**		
**Samples**	**25°C**	**40°C**	**50°C**
**Bulk CH_**3**_OH**	**2,800 ± 20 ms**	**3,300 ± 100 ms**	**3,320 ± 20**
150 mg silica-4.0 nm + 0.9 ml CH_3_OH	(1): 50 ± 30 ms	(1): 200 ± 40 ms	(1): 520 ± 70 ms
	(2): 540 ± 10 ms	(2): 670 ± 20 ms	(2): 900 ± 100 ms
150 mg silica-4.0 nm + 0.6 ml CH_3_OH	(1): 100 ± 10 ms	(1): 100 ± 30 ms	(1): 150 ± 20 ms
	(2): 465 ± 6 ms	(2): 530 ± 10 ms	(2): 630 ± 30 ms
150 mg silica-4.0 nm + 0.4 ml CH_3_OH	(1): 120 ± 20 ms	(1): 40 ± 20 ms	(1): 140 ± 30 ms
	(2): 400 ± 21 ms	(2): 390 ± 10 ms	(2): 470 ± 20 ms
**Bulk CH**_**3**_**OD**	**2,790 ± 30 ms**	**3,170 ± 50 ms**	**3,280 ± 40 ms**
150 mg silica-4.0 nm + 0.9 ml CH_3_OD	(1): 1,290 ± 10 ms	(1): 1,560 ± 30 ms	(1): 1,300 ± 200 ms
			(2): 2,000 ± 200 ms
150 mg silica-4.0 nm + 0.6 ml CH_3_OD	(1): 100 ± 20 ms	(1): 780 ± 20 ms	(1): 680 ± 80 ms
	(2): 670 ± 6 ms		(2): 1,000 ± 100 ms
150 mg silica-4.0 nm + 0.4 ml CH_3_OD	(1): 390 ± 40 ms	(1): 580 ± 20 ms	(1): 650 ± 20 ms
	(2): 630 ± 60 ms		

**Table 5B:** Transverse magnetization relaxation times (T_2_) of confined CH_3_OH obtained after sonication of the mixtures for 15 min.

	**Temperature (°C)**		
**Samples**	**25°C**	**40°C**	**50°C**
Bulk CH_3_OH	2,589.5 ± 0.1 ms	2,780.9 ± 0.2 ms	2,985.6 ± 0.2 ms
150 mg silica-4.0 nm + 0.9 ml CH_3_OH	(1): 59.1 ± 0.8 ms	(1): 61.9 ± 0.6 ms	(1): 53.0 ± 0.3 ms
	(2): 338.5 ± 0.4 ms	(2): 396.1 ± 0.3 ms	(2): 434.5 ± 0.3 ms
150 mg silica-4.0 nm + 0.6 ml CH_3_OH	(1): 49.9 ± 0.5 ms	(1): 44.2 ± 0.5 ms	(1): 39.1 ± 0.3 ms
	(2): 307.3 ± 0.4 ms	(2): 327.1 ± 0.4 ms	(2): 386.2 ± 0.3 ms
150 mg silica-4.0 nm + 0.4 ml CH_3_OH	(1): 32.1 ± 0.4 ms	(1): 28.2 ± 0.3 ms	(1): 23.2 ± 0.2 ms
	(2): 171.3 ± 0.4 ms	(2): 196.1 ± 0.3 ms	(2): 209.8 ± 0.3 ms
Bulk CH_3_OD	2,588.6 ± 0.2 ms	2,790.3 ± 0.4 ms	2,937.3 ± 0.2 ms
150 mg silica-4.0 nm + 0.9 ml CH_3_OD	(1): 58 ± 2 ms	(1): 64 ± 2 ms	(1): 38 ± 1 ms
	(2): 636.1 ± 0.3 ms	(2): 732.8 ± 0.3 ms	(2): 807.2 ± 0.2 ms
150 mg silica-4.0 nm + 0.6 ml CH_3_OD	(1): 48 ± 1 ms	(1): 145 ± 3 ms	(1): 32.7 ± 1.0 ms
	(2): 422.0 ± 0.3 ms	(2): 521.5 ± 0.8 ms	(2): 527.5 ± 0.2 ms
150 mg silica-4.0 nm + 0.4 ml CH_3_OD	(1): 58 ± 2 ms	(1): 59 ± 3 ms	(1): 50 ± 2 ms
	(2): 231.9 ± 0.6 ms	(2): 259.2 ± 0.8 ms	(2): 273.4 ± 0.5 ms

**Figure 5:**
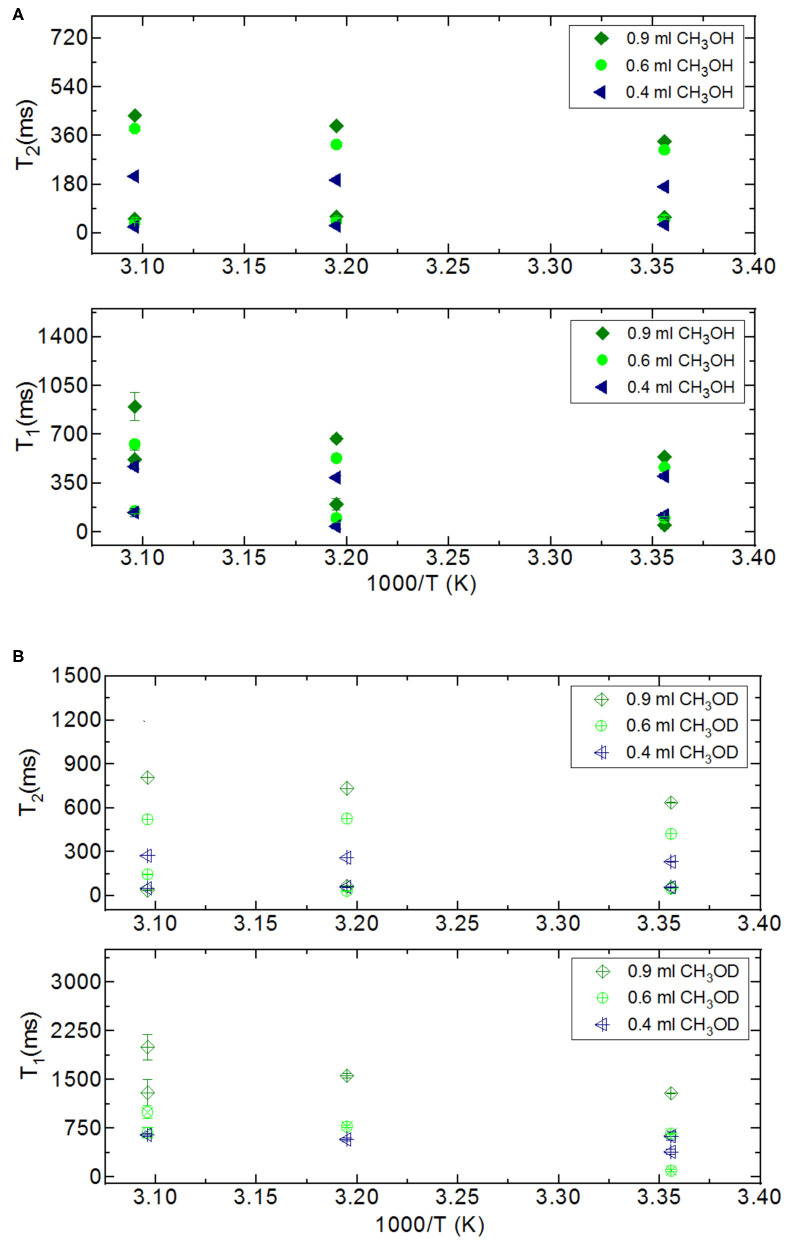
Comparison of relaxation values of CH_3_OH and CH_3_OD confined into silica-4.0 nm with the amount of 150 mg and S/V = 574 where the variables are temperature values (298, 313, and 323 K), and fluid-to-solid ratios (0.90 ml/150 mg, 0.60 ml/150 mg, 0.40 ml/150 mg); CH_3_OH **(A)**, CH_3_OD **(B)**.

Methanol has also two T_2_ values in confined state (Figures 5A,B). The longer T_2_ value is greater than of that H_2_O. The longer T_2_ value increases as temperature increases, while the shorter T_2_ does not indicate significant change when temperature is varied. When the fluid-to-solid ratio was decreased, T_2_ values also decreased. The short T_2_ value of CH_3_OH in the confined state became even shorter as the temperature was elevated. This is independent of the fluid-to-solid ratios. This observation is explained by layering of CH_3_OH on the pore walls via interacting with –OH groups of the pores, which are proven to exist by solid-state magic angle spinning (MAS) cross-polarization (CP) NMR (Sindorf and Maciel, 1983). In addition, water molecules near the interface had preferred directions due to the interaction with surface –OH groups (Millischuk and Ladanyi, 2011). There are two layers, and as temperature was elevated the molecules of the second layer having weaker interactions with the molecules forming the first layer with –OH of the pore walls gained mobility, and moved to the region of the molecules clustered in the middle of the pores. The number of methanol molecules in the second layer was reduced when the temperature was increased, while methanol molecules of the first layer still interact with the pore walls. Thus, the shorter T_2_ value attributed to the average motions of the first and second layers of CH_3_OH interacting with the pore walls became shorter at high temperatures. However, there is weaker hydrogen bonding interaction between CH_3_OH and –OH of the pore walls that is indicated in relatively longer T_1_ values of CH_3_OH with respect to these of H_2_O. As discussed above, the contribution of faster internal –CH_3_ rotation to the overall dynamics of CH_3_OH molecules should not be neglected as well.

### Dynamics of Water Confined in Nanoporous Silica Monoliths Without Excess Fluid

**Figure 6:**
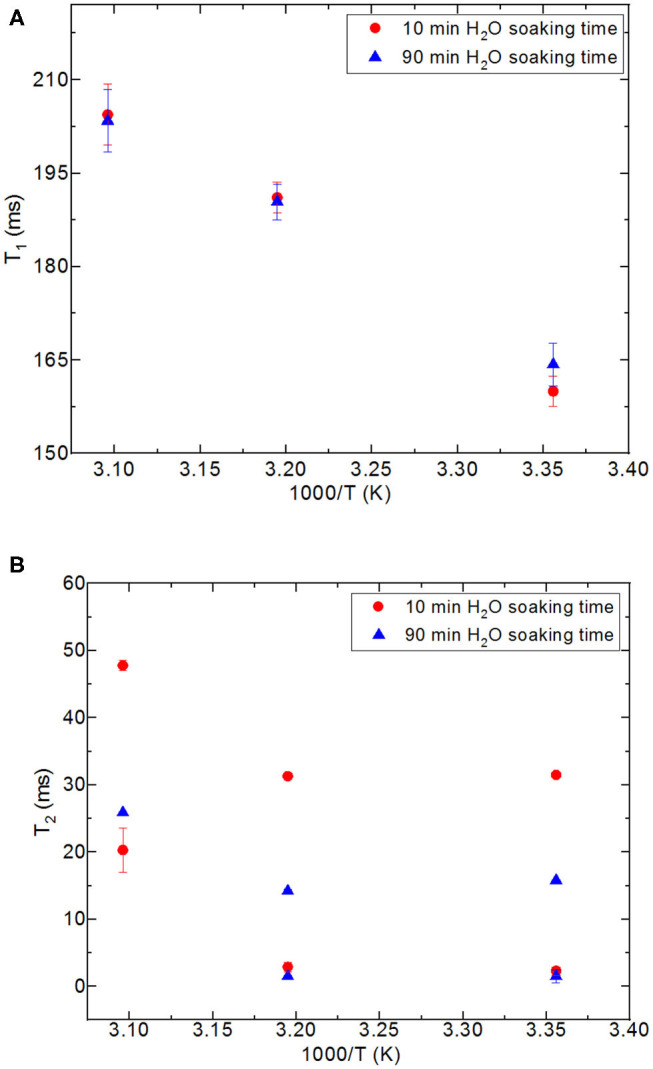
Comparison of relaxation values of H_2_O confined into 6 nm silica rod at 298, 313, and 323 K, and with 10 and 90 min of water soaking times; T_1_ **(A)**, T_2_ **(B)**.

First, it needs to be mentioned that there was no excess fluid in the medium when both of the T_1_ and T_2_ measurements were conducted upon soaking the nanoporous silica monoliths into water. The surface area of the nanoporous silica monolith is closer to that of silica-4.0 nm. Table 6 lists T_1_ and T_2_ values of water confined in the nanoporous silica monolith by varying soaking duration and temperature (see Figure 6). In this case, 10 min and 90 min of soaking time correspond to partial filling and more complete saturation of water in pores, respectively. Both T_1_ and T_2_ times of confined water in the nanoporous silica monoliths show strong deviation from bulk values (see Tables 4A,B) for bulk T_1_ and T_2_ relaxation values of water, respectively). The deviation from bulk is independent of the water soaking time. Comparison of the T_1_ values water molecules inside the nanoporous silica monoliths shows the negligible effect of soaking duration. However, T_2_ times get shorter approximately twice as the soaking time was increased to 90 min. As expected, when the temperature was increased, both T_1_ and T_2_ times of confined water within the nanoporous silica monoliths get longer, and again independent of the soaking time. The shortest T_2_ value observed is explained by the strong interaction between the confined water molecules and the pore walls of the nanoporous silica monolith surface. We suggest that there is first a monolayer established on top of the pore walls of the monolith. This layer gains mobility when the temperature was increased in the case of short soaking time. However, such a mobility and hence increase in the shorter T_2_ time component is not observed in the case of 90 min soaking time sample. In other words, partial filling of the pores due to shorter soaking time gives free volume within the nanoporous silica monolith for confined water molecules. The elevation in temperature helps with overcoming energy barrier that water molecules forming the monolayer gain freedom. This gain in mobility is reflected as longer relaxation times. In comparing the relaxation times of confined water in nanoporous silica monoliths with respect to the relaxation values of H_2_O confined into nanoporous powdered silica samples (silica-4.0 nm, silica-2.5 nm, and silica-1.5 nm), we prefer to consider the samples where only 0.4 ml water was confined into the powdered silica samples. T_1_ values of water within the nanoporous silica monoliths are significantly shorter than those of water confined into silica-2.5 nm and silica-1.5 nm, while closer but still shorter than that of water confined into silica-4.0 nm. T_2_ times of water confined into nanoporous silica monolith for 10 min of soaking time are longer than those of T_2_ times of water confined into powdered nanoporous silica samples, but T_2_ times of water confined into nanoporous silica monolith for 90 min of soaking time are within the same range with T_2_ values of water confined into powdered nanoporous silica matrixes. The comparison shows that confinement effect for water is independent of water soaking time. The water molecules remaining between powdered nanoporous silica grains upon sonication, where only 0.4 ml water was confined into the powdered silica samples, contribute to the averaged relaxation times so that relatively longer T_1_ and T_2_ times than those of water within the nanoporous silica monoliths were observed. There is an overall decreasing trend in relaxation times of water as going from smaller pore diameters of 1.5 and 2.5 nm to larger diameters of 4.0 and 6.0 nm. As a result, we suggest that taking the pore diameters of both the powdered and monolith samples into account, pore volume is a significant factor reducing the mobility of water.

**Table 6:** T_1_ and T_2_ values of water confined into nanoporous silica rod.

**Silica_rod_6 nm**			**Silica_rod_6 nm**		
**Water soaking time 10 min**			**Water soaking time 90 min**		
**Temperature (°C)**	**T_**1**_ (ms)**	**T_**2**_ (ms)**	**Temperature (°C)**	**T_**1**_ (ms)**	**T_**2**_ (ms)**
25	160 ± 2.4	31.5 ± 0.2	25	164.3 ± 3.4	15.8 ± 0.1
		2.3 ± 0.6			1.5 ± 1.0
40	191.1 ± 2.5	31.3 ± 0.2	40	190.4 ± 2.9	14.2 ± 0.2
		2.9 ± 0.7			1.5 ± 0.0
50	204.4 ± 4.9	47.8 ± 0.8	50	203.4 ± 5.0	25.89 ± 0.1
		20.3 ± 3.3			

### T_1_/T_2_ Ratio of Confined Fluid Interaction With the Pore Walls

Regarding NMR relaxation in porous media, T_1_/T_2_ ratio indicates how strongly or weakly molecules interact with the uppermost layer of the porous system (Mitchell et al., 2009; Weber et al., 2009). Therefore, T_1_/T_2_ ratio is an important parameter showing the degree of wettability of the fluid toward pore walls in confined geometry. The NMR T_1_ time in liquids arises from time-dependent local magnetic fields persuading changeovers that permit nuclear spins to restore the equilibrium. The major driving force for variations in local magnetic fields at a nucleus is the rotational motion of molecules, regularly defined as molecular tumbling. The correlation time of molecular tumbling is defined as τ_c_. The approximate definition of τ_c_ is the duration for a molecule to finish a rotation of 1 rad. The BPP (Bloemberg-Purcell-Pound) relaxation theory describes that for τ_c_ → 0, T_1_ → ∞. As a result, small, and rapid tumbling molecules will show a gradual relaxation rate and thus longer T_1_ values (Bloembergen et al., 1948; D'Agostino et al., 2012). There is the resulting correlation between T_1_ and τ_c_:

(3) $$\begin{array}{r} \frac{1}{T_{1}}\propto \frac{\tau_{c}}{1+{\left(2\omega_{0}\tau_{c}\right)}^{2}} \end{array}$$

where ω_0_ is the precession frequency of the target nucleus. This equation tells that for very fast molecular motion (when 1/τ_c_ >> 2ω_0_), T_1_ is inversely proportional to τ_c_. That is, as correlation time decreases (fast molecular motion), relaxation time increases (the rate of relaxation decreases). Conversely, for slow molecular motion (when 1/τ_c_ << 2ω_0_), T_1_ is directly proportional to τ_c_. Therefore, they both increase together. The minimum in T_1_, and hence the most efficient spin-lattice relaxation, occurs when τ_c_ α (ω_0_)^−1^ (Slichter, 1990; Macomber, 1998; Nicotera et al., 2012). Viscous molecules or molecules under confinement of geometrical constraints will have shorter T_1_ values and thus a faster relaxation rate (D'Agostino et al., 2012). In analyzing and explaining the longitudinal relaxation data obtained, we refer to Equation (4) described below. We first think of a cylindrical pore with a radius of R and a length of l, completely filled with water molecules. Due to water-substrate interactions, the water molecules at the surface will experience a restricted motion when compared to the bulk like water in the middle of pore. We assume that the water molecules within a distance *a* from the surface are affected by the surface and the rest acts as bulk like fluid as mentioned above while discussing layering of methanol on the pore walls via interactions with –OH groups decorating the pore walls. Under these assumptions the observed proton relaxation time constant T_1,observed_ will have a weighted average from water molecules near the surface and bulk like water molecules, where the weights are proportional to the volume (Gallegos et al., 1988; Weber et al., 2009). The number of protons/water molecules at the uppermost layer and in bulk-like state is proportional to the V_Surface_ and V_Bulk_, respectively. Thus, the observed T_1,observed_ relaxation can be written as:

(4) $$\begin{array}{r} \frac{1}{T_{1,observed}}=\frac{2a}{R}\left[\frac{1}{T_{1,surface}}-\frac{1}{T_{1,bulk}}\right]+\left[\frac{1}{T_{1,bulk}}\right] \end{array}$$

This equation assumes an ideal case where the pores are filled in whole with the liquid of interest. In the current study, there is gradual increase in the amount of fluid mixed with the porous systems. This leads to an increase in the amount of confined fluid. Tables 7A,B lists the T_1,surface_ values, where T_1,surface_ was calculated by the following restrictions: (i) only 0.4 ml fluid added samples are taken into consideration, (ii) for *a*, 0.25 nm as the silica layer thickness is assumed. As shown in Table 7A, T_1,surface_ values decreased as the temperature was increased only in the case of silica-4.0 nm and silica-1.5 nm. On the contrary, in the case of silica-2.5 nm, T_1,surface_ values increased as the temperature was increased. This shows that water molecules form layers on the pore walls of silica-4.0 nm and silica-1.5 nm, and these layers have strong interaction with the pore walls. The contrary dynamical attitude of water in silica-2.5 nm is a reflection of the effect of surface-to-volume ratio. Table 7B shows that T_1,surface_ values of CH_3_OH in the nanopores of silica-4.0 nm do not change significantly as a function of temperature. However, there is a systematic increase in T_1,surface_ values of CH_3_OD in the nanopores of silica-4.0 nm when the temperature was elevated. This is attributed to the fluid chemistry.

**Table 7A:** T_1,surface_ values of water in confined state.

	**T_**1,surface**_ (ms) with 0.4 ml water**		
	**25°C**	**40°C**	**50°C**
Silica-4.0 nm	28.0	47.4	3.8
Silica-2.5 nm	842.7	909.4	1066.9
Silica-1.5 nm	263.0	182.0	44.2

**Table 7B:** T_1,surface_ values of CH_3_OH and CH_3_OD in confined state.

	**T_**1,surface**_ (ms): silica-4.0 nm + 0.4 ml fluid**		
	**25°C**	**40°C**	**50°C**
CH_3_OH	15.6	5.1	18.2
CH_3_OD	55.5	86.3	98.3

Sattig et al. (2014) suggested that ^2^H NMR line-shape analysis evidenced pronounced dynamical heterogeneities for confined H_2_O. In another study on characteristic properties of H_2_O dynamical attitudes in confined forms explored by quasi-elastic neutron scattering, Osti et al. (2016) mentioned primary single parameter θ, which is the ratio of the mean number of water molecules that are mostly affected by pore walls to the total number of H_2_O molecules under confinement. If we consider θ as equal to a constant value such as (X) for a complete saturation with and without excess water by taking filling mechanism of MCM-41 with H_2_O mentioned by Grünberg et al. (2004) into account, we suggest that there are both θ ≈ X and θ ≤ X where there is dynamic exchange between confined water molecules (the ones not interacting with the pore walls) and the bulk excess water. For the samples with excess fluid as in the case of silica-4 nm, θ for different fluid-solid-ratios is equal to each other. When the fluid-to-solid ratio is decreased for the partially filled samples, θ also decreases down. Because θ is utilized for the slit-type, cylindrical, and spherical geometries, θ is applicable in the current study as well. Osti et al. (2016) used exact analytical techniques to explain H_2_O adsorption in three model geometries of slit-type, cylinder, and sphere to have deeper explanation of the change of θ with the restricted geometry. In the present study, characterization of the engineered nanoporous proxies and the analysis of confined fluids are done in detail. For example, although nanoporous silica materials are known amorphous, degree of regular patterns of each sample was studied by XRD thoroughly to clarify the alignment and geometry of the pores.

A rough consideration of T_1_/T_2_ ratios for both confined water and methanol clearly indicate a straightforward deducing arrangement of comparative intensity of interaction with the pore uppermost layer of silica-4.0 nm: water has greater interaction with the pore surface than methanol (Weber et al., 2010; D'Agostino et al., 2012). For this reason, we think that methanol molecules establish clusters with extensive hydrogen bond formation in the pores rather than interacting with the pore surface (Tsotsalas et al., 2013). It was argued that the existence of porous medium disrupts the widespread intermolecular hydrogen-bonding system of some polyols such as glycerol and ethylene glycol, and this disruption in hydrogen bonding system resulted in boosted translation and tumbling movement degree, therefore longer T_1_ values. In our case, such a disruption might exist with respect to bulk liquids. However, this disruption is less effective for confined CH_3_OH molecules than confined water molecules given that the same nanoporous matrix system is utilized for confining the molecules. In addition, we observe shorter T_1_ times for both of the confined fluids with respect to their bulk state. It is already known that NMR relaxation time values of confined fluids in porous media are relatively shorter than their corresponding fluids in bulk (Barrie, 2000). This situation is attributed to the uppermost layer and dipolar interactions and a decreased degree of reorientation of molecules at the pore uppermost layer (D'Agostino et al., 2012). We suggest these interactions dominate in terms of reducing the T_1_ times of both of the confined fluids.

In addition to comparing relaxation behaviors of confined H_2_O and CH_3_OH, we also compare relaxation behaviors of CH_3_OH and CH_3_OD under confinement. When we switch from CH_3_OH to CH_3_OD, we only measure confined behavior of –CH_3_ group. Both T_1_ and T_2_ of CH_3_OD are longer than those of CH_3_OH. T_1_/T_2_ ratio of confined CH_**3**_OD is also smaller as in the case of confined CH_3_OH. This shows that there is weaker interaction between –CH_3_ and mesoporous silica-4.0 nm. Although confined CH_3_OD behavior deviated from that of bulk CH_3_OD upon confining into silica-4.0 nm, deviation is lesser than these of confined H_2_O and CH_3_OH. This also shows that hydrogen bonding capability of the molecules such as H_2_O and CH_3_OH with –OH groups decorating the pore walls influences both dynamics of the confined fluids and the layers formation on the pore walls.

### T_2_ Distributions of Fluids Under Nanoporous Confinement

**Figure 7:**
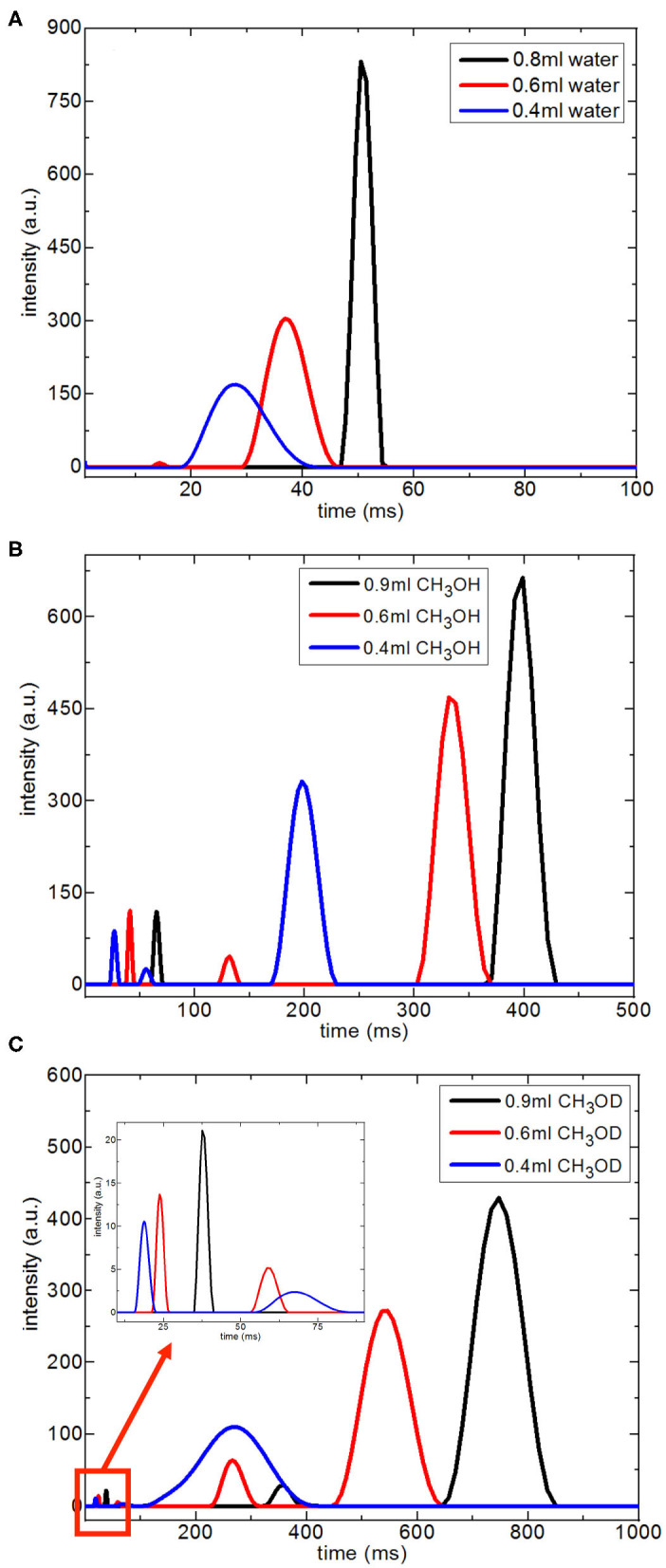
Continuous T_2_ relaxation time data for H_2_O **(A)**, CH_3_OH **(B)**, and CH_3_OD **(C)** confined into silica-4.0 nm with the amount of 150 mg and S/V = 574 as a function of fluid-to-solid ratio.

Figure 7 shows T_2_ distribution curves, obtained using Contin software, of H_2_O, CH_3_OH, and CH_3_OD confined in silica-4.0 nm. The distributed exponential approach utilizes a governing approach to the inverse Laplace transform yielding a continuous distribution of T_2_ relaxation times (Provencher, 1982). Mathematically speaking, the distributed exponential fitting is an ill-defined process because it is sensitive to the restrictions applied (Martens and Thybo, 2000; Hansen et al., 2010). Furthermore, there is a shorter T_2_ relaxation component observed in the range 10–15 ms. Such a component is mostly explained as a treating artifact due to the Contin processing stage (Aursand et al., 2008). For this reason, this component in the T_2_ distributions is not taken into account in evaluating T_2_ distribution curves of the fluids confined into silica-4.0 nm.

In the case of the confined water inside silica-4.0 nm, when the fluid-to-solid ratio is lowered, T_2_ curves shifted toward shorter times. In addition, T_2_ curves became broader and had lower intensity (Figure 7A). On the other hand, confined CH_3_OH has longer T_2_ components as seen in Figure 7B. The T_2_ curves with the highest intensities between 150 and 450 ms range exhibited the same tendency as in the case of confined water. In other words, lower fluid-to-solid ratio was reflected as lower intensity T_2_ distribution curves at shorter T_2_ times. For the range between 20 and 80 ms the same result was observed. However, line broadening in T_2_ distribution curves of confined CH_3_OH was less pronounced. When the fluid-to-solid ratio was at the lowest value (0.4 ml CH_3_OH confined to 150 mg silica 4.0 nm), there was a third T_2_ curve around 55 ms with broader distribution. When the fluid chemistry was switched to CH_3_OD, T_2_ distribution curves with the higher intensities were in the range between 100 and 900 ms. Consistently with the two other confined fluids, as the fluid-to-solid ratio was decreased, T_2_ curves shifted to shorter times with lower intensities. However, there were three T_2_ curves for each fluid-to-solid ratio. As the T_2_ curve shifts toward lower values, the T_2_ curve loses intensity. For this reason, the observation of three T_2_ curves for confined CH_3_OD inside silica-4.0 nm might correspond to different proportions of the confined CH_3_OD molecules (Bertram et al., 2002): (1) the ones forming layers on the pore walls by weak interactions with the –OH of the pore wall, (2) the ones closer to the middle of the pores and having lesser interactions with ones forming layers on the pore walls, (3) the ones clustering in the center of the pores. As reflected in longer T_2_ values, and continuous T_2_ distribution curves, among the three fluids of interest, CH_3_OD has the weakest interaction with the pore walls.

## Conclusion

Specific pore volumes (cm^3^/g) of the nanoporous silica samples studied exhibit the following order: silica-1.5 nm > silica-4.0 nm > silica-2.5 nm. Nanopore diameter does not influence the dynamics of confined water significantly as revealed by the results on relaxation of water obtained by keeping pore volumes of porous matrixes constant for the given amounts of the porous materials. Rather than pore diameter, S/V ratio along with pore diameter reflects information on filling mechanism the nanopores with water. In the case of relatively larger pore diameter and small S/V ratio, wetting the surface of the nanopore walls is followed by additional filling from the pore wall to the midpoint of the pore until achieving complete filling of the pores (Grünberg et al., 2004). In the case of nanoporous silica with small pore diameter and large S/V ratio, the filling mechanism follows a different path: first, fluid fills the center of the pores leading to a co-presence of filled pore fragments with wetted pores. Further filling of the pores develops axially in the direction of the pore axis.

The possibility of distinguishing among relaxation times of excess and confined fluids, and to quantify excess fluid, fluid found in the interparticle regions of nanoporous silica powder and outside the pores, and fluid confined in the nanopores of silica matrices might have applications in the petroleum industry, where characterization of rock with various pore sizes has significance for better definition of reservoirs. However, the highest S/V ratio leads to the longest T_1_ times of confined water, while the lowest S/V ratio resulted in the shortest T_1_ times of confined water. The decrease in T_2_ values as decreasing the fluid-to-solid ratio is more pronounced in the case of silica-2.5 nm having the lowest pore volume. These results clearly show the importance and influence of S/V ratio on dynamical behaviors of fluids confined into nanoporous proxies.

Comparison of T_1_/T_2_ ratios, showing the affinity of the confined fluid to the pore walls, of both confined H_2_O and CH_3_OH clearly show that H_2_O has stronger interaction with the pore surface of silica-4.0 nm than CH_3_OH. The degree of deviation of confined CH_3_OD behavior in silica-4.0 nm from that of bulk is less than those of confined H_2_O and CH_3_OH. It was claimed that a porous medium disrupts the extensive intermolecular hydrogen-bonding network of some polyols, and this disruption led to longer T_1_ values (D'Agostino et al., 2012). Such a disruption in hydrogen-bonding network is less effective for confined CH_3_OH molecules than confined H_2_O molecules for the same nanoporous matrix system. In addition to S/V ratio, chemistry of confined fluids also affects the dynamical measurements. This is an important observation, for example, in interpreting dynamical behaviors of confined and saturated mixtures of water and hydrocarbons at natural confined systems, where wettability alteration occurring inside the porous structures of the rocks influences the crude oil production.

The continuous T_2_ distribution curves suggest that weaker interaction between CH_3_OD and nanoporous silica-4.0 nm with three different environments: (i) layered structure by weak interactions with the pore walls, (ii) the ones closer to the center of pores and interacting weakly with the first layer on the pore wall, (iii) the ones forming cluster in the middle of the nanopores.

Finally, the characteristics and properties of the nanoporous matrix systems are defined in a better way. Furthermore, low-field ^1^H NMR is a powerful technique when exploring confined fluid dynamics as a function of the aforementioned parameters. These nanoporous silica samples are ideal examples that mimic the natural geological systems such as heterogeneous subsurface materials containing crude oil and brine solutions to mimic nano-environments present in natural systems such as rocks. Based on the findings on the properties of mesoporous silica proxies and dynamical behavior of confined crude oil and brine confined into mesoporous silica materials, we also analyzed sandstone rock cores flooded with crude oil and brine with hydrophobic/oleophilic nanoparticles in addition to characterizing natural carbonate rock cores saturated with water and crude oil. The results of these measurements are to be discussed in forthcoming publications.

## Data Availability Statement

All datasets generated for this study are included in the article/Supplementary Material.

## Author Contributions

SO planned and conducted the low-field NMR measurements in addition to TGA measurements. BH contributed to the low-field NMR measurements. TL completed the BET and TXRD measurements. JS and SW contributed to each and every step of the study, and in particular on both TXRD and BET parts. K-HL and C-YM synthesized silica-2.5 nm and silica-1.5 nm. DC was the research leader. All authors contributed to the article and approved the submitted version.

## Conflict of Interest

The authors declare that the research was conducted in the absence of any commercial or financial relationships that could be construed as a potential conflict of interest.
