# Supplementary material for: Fluid Behavior in Nanoporous Silica
Source: Front Chem. 2020 Aug 28;8:734. doi: 10.3389/fchem.2020.00734 (PMC7485247; doi:10.3389/fchem.2020.00734)
Supplement: Supplementary file 1 [file Data_Sheet_1.docx]

SUPPLEMENTARY INFORMATION FOR

**Fluid Behavior in Nanoporous Silica**

Salim Ok^1,*^, Bohyun Hwang^1^, Tingting Liu^1^, Susan Welch^1^, Julia Sheets^1^, David R. Cole^1, 2^, Kao-Hsiang Liu^3^, Chung-Yuan Mou^4^

*^1^School of Earth Sciences and ^2^Department of Chemistry, The Ohio State University, Columbus, Ohio 43210*

*^3^*Joint Institute for Neutron Science *Oak Ridge National Laboratory* Oak Ridge, Tennessee 37831, USA

*^4^Department of Chemistry, National Taiwan University, No. 1, Sec. 4, Roosevelt Road, Taipei, 10617 Taiwan (R.O.C.)*

***Corresponding author**

**Contents**

1. **TABLE S1**. Surface areas, volumes, and sizes of the pores of model mesoporous silica materials.
2. **Figure S1**. Nitrogen adsorption and desorption isotherms at 77 K for the three materials in this study. The insets are the pore size distribution plots, derived from different models.
3. **Figure S2 (a-c)**. TXRD patterns of (a) silica-4.0 nm, (b) silica-2.5 nm, and (c) silica-1.5 nm in this study.
4. **Figure S3 (a-b)**. Representative TGA curve of the engineered silica-4.0 nm (similar results were observed in the cases of silica-2.5 nm and silica-1.5 nm) (a), mass loss of the silica samples as a function of drying time revealing pore volume capacity of the silica samples (b).
5. **Figure S4 (a-e)**. The mixture of 150 mg silica-4.0 nm and 0.8 ml water with excess fluid on top after centrifugation (a), and after homogenization by sonication for 15 minutes (b), no excess water was observed in the mixture of 150 mg silica 2.5 nm and 0.8 ml water after centrifugation (c), the mixture of 150 mg silica 1.5 nm and 0.8 ml water with excess fluid on top after centrifugation (d), and after homogenization by sonication for 15 minutes (e). The “object” behind the vials is utilized to show the “excess water” on top of the vials in images (a) and (d) versus “cloudy” situation on top of the vials in images (b), (c), and (e).

**TABLE S1.** Surface areas, volumes, and sizes of the pores of model mesoporous silica materials.

|  | Silica-4.0 nm | Silica-2.5 nm | Silica-1.5 nm |
| --- | --- | --- | --- |
| BET Surface area (m^2^/g) | 597 | 1167 | 832 |
| Pore Volume (cm^3^/g), single point at P/P_0_=0.99 | 1.04 | 0.97 | 1.20 |
| Pore Size (Å), BJH desorption model | 42 | 28 | 15, 22 |
| Surface area/Volume (S/V) | 574 | 1203 | 693 |

**Figure S1.**

**Figure S1**. Nitrogen adsorption and desorption isotherms at 77 K for the three materials in this study. The insets are the pore size distribution plots, derived from different models.

**Figure S2 (a-c).**

**Figure S2 (a-c)**. TXRD patterns of (a) silica-4.0 nm, (b) silica-2.5 nm, and (c) silica-1.5 nm in this study.

**Figure S3 (a-b).**

**(a)**

**(b)**

**Figure S3 (a-b)**. Representative TGA curve of the engineered silica-4.0 nm (similar results were observed in the cases of silica-2.5 nm and silica-1.5 nm) (a), mass loss of the silica samples as a function of drying time revealing pore volume capacity of the silica samples (b).

**Figure S4 (a-e).**


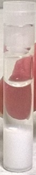

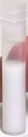

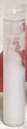

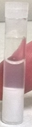
 **
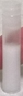
**

**(a) (b) (c) (d) (e)**

**Figure S4 (a-e)**. Sample images used in NMR experiments: the mixture of 150 mg silica-4.0 nm and 0.8 ml water after centrifugation (a), and after homogenization by sonication for 15 minutes (b), no excess water observed in the mixture of 150 mg silica 2.5 nm and 0.8 ml water after centrifugation (c), the mixture of 150 mg silica 1.5 nm and 0.8 ml water after centrifugation (d), and after homogenization by sonication for 15 minutes (e). Object behind vials is utilized to show the “excess water” at the top of vials in (a) and (d) in contrast to turbid fluid in (b), (c), and (e).
